# The Impact of Ovulation Calculators on the Stress Levels of Fertility-Seeking Couples: An Evaluation Study

**DOI:** 10.7759/cureus.43972

**Published:** 2023-08-23

**Authors:** Braian R Ledesma, Maria Camila Suarez Arbelaez, Meghan Grewal, Kyara Marquez, Katherine Palmerola, Armin Ghomeshi, Ranjith Ramasamy

**Affiliations:** 1 Urology, Desai Sethi Urology Institute, University of Miami, Miami, USA; 2 Urology, Jackson Health System, University of Miami, Miami, USA; 3 Obstetrics and Gynecology, IVFMD, Miami, USA; 4 Urology, Florida International University Herbert Wertheim College of Medicine, Miami, USA

**Keywords:** sexual dysfunction, andrology, stress, ovulation calculator, infertility

## Abstract

Introduction

Infertility affects a significant portion of couples seeking pregnancy, leading to stress and emotional strain. Ovulation calculators, widely used as a tool to predict fertile days, may play a role in the stress experienced by couples undergoing fertility treatments. The aim of this study was to evaluate the impact of ovulation calculators on the stress levels of couples seeking fertility.

Methods

Participants were recruited from the University of Miami Health System Clinics. Fifty couples consulting for infertility were asked to participate in the study and complete anonymous self-reported surveys. The surveys consisted of validated questions related to stress levels and the use of ovulation calculators. The completed surveys were collected and analyzed using descriptive statistics. The data collected included age, number of years trying to conceive, and answers to questions related to stress levels and the use of ovulation calculators. Responses from 50 couples who met the inclusion criteria were included in the final analysis.

Results

A total of 50 couples who were attempting conception and who completed all the questionnaires were included in the study. Whether or not they were using ovulation calculators, females scored similarly in the four variables of the Female Sexual Function Index (FSFI), including arousal, orgasm, satisfaction, and lubrication. When evaluating International Index of Erectile Function (IIEF) scores for male erectile function, the average score of males tracking ovulation was 12.0 ± 4.8, compared to 11.5 ± 5.4 in male patients who were not (P = 0.81). The results showed no statistically significant difference in stress levels between couples who used ovulation calculators and those who did not. However, in couples experiencing higher stress levels due to infertility, both male and female participants reported higher levels of sexual dysfunction. Fertility-related stress was also found to be significantly associated with mental health implications, with increased anxiety and depression reported by couples undergoing fertility treatments.

Conclusion

The findings suggest that the use of ovulation calculators did not significantly influence the stress experienced by couples seeking fertility treatment. However, the study highlights the significant impact of infertility-related stress on sexual function and mental health in both male and female partners. These results emphasize the importance of addressing the psychological aspects of infertility and providing comprehensive support to couples undergoing fertility treatments. Further research is warranted to explore the complex interplay between ovulation calculator usage, infertility-related stress, sexual dysfunction, and mental health implications in couples seeking to conceive. Healthcare providers should consider incorporating mental health support into fertility treatment programs to optimize patient outcomes and overall well-being.

## Introduction

As per the World Health Organization's statistics, around 48 million couples encounter infertility, which is characterized by the inability to achieve clinical pregnancy following a year of consistent, unprotected sexual intercourse or a hindrance in an individual's ability to reproduce, either independently or with a partner. This issue appears to be on a persistent rise [1,2]. Failure to conceive and coping with infertility can have a profound impact on couples, often reported as major life stressors, significantly affecting both the physical health and emotional well-being of many individuals [3,4].

Among couples trying to conceive, ovulation tests are a simple, effective, and popular method to maximize the chances of a natural conception [3]. Conception most often occurs during a six-day fertile window, which ends on the day of ovulation. Timed intercourse (TI) during this period is one of the simplest and most commonly prescribed treatments for couples who want to become pregnant [3]. Implementing ovulation calculators can help prospectively identify a female's most fertile phase. Through this method of family planning, known as fertility-focused timed intercourse, conception is likely to occur more quickly [5]. Increased ability to identify the fertile phase can help females achieve pregnancy sooner by allowing synchronized intercourse with fertility phases.

Despite the advantages and positive outcomes that ovulation tests provide, coordinating sexual intercourse in this way has been associated with increased emotional distress [6]. While sexual function is an important component of health, it is a frequently compromised aspect of quality of life in couples affected by infertility. In general, current data reveals that about 40%-45% of adult females and 20%-30% of adult males have at least one manifestation of sexual dysfunction; these figures may be higher in people experiencing infertility and even more so in those experiencing infertility and implementing timed intercourse, which can add to sexual difficulties [4]. Similarly, repeated unsuccessful conceptions, despite strict enforcement of timed intercourse, can lead to anxiety and contribute to the worsening of the mental and emotional well-being of both partners. Current clinical guidelines released by the National Institute for Health and Care Excellence (NICE) state that the timing of intercourse to coincide with ovulation causes stress and is not recommended [7].

The International Index of Erectile Function (IIEF) is a widely used patient-reported outcome measure that addresses the relevant domains of male sexual function, including erectile and orgasmic function, sexual desire, intercourse satisfaction, and overall satisfaction [7,8]. Given its strong psychometric properties and ability to provide a quantitative index of the erectile dysfunction (ED) severity for diagnostic and classification purposes, the IIEF has become a widely adopted clinical resource for the evaluation of male sexual function; however, no studies, to our knowledge, have used the IIEF to demonstrate the effects of infertility stressors on male sexual function. Similarly, the Female Sexual Function Index (FSFI) scale comprises six domains: desire, arousal, lubrication, orgasm, satisfaction, and pain. The FSFI has been shown to have good psychometric properties and has been used to assess sexual function in females. Despite its numerous implementations, the FSFI has not been implemented to directly assess and measure the effects of infertility stressors on female sexual function [9].

Many validated questionnaires have been developed to provide insight into perceived infertility-related stress. One reliable self-reported questionnaire is the Fertility Problem Inventory (FPI), an instrument that identifies and measures important domains of stress specific to infertility [10]. The FPI focuses on five distinct domains and categories of origin of stress in couples facing infertility: social concern, sexual concern, relationship concern, need for parenthood, and rejection of a child-free lifestyle [10]. The FPI has revealed heightened dissatisfaction with sexual relations that require scheduling, a factor that was also conjectured to contribute to sexual dysfunction; however, given the comprehensive nature of the questionnaire and the considerable overlap that exists between the different constructs, this data cannot be explicitly applied to a single domain nor used to provide an understanding of the sexual concerns that arise from infertility-related stress [10].

Despite these resources for assessing sexual function, current findings are limited to the association between infertility-related stress and sexual dysfunction. Specifically, little is known regarding the association between ovulation test use and stress levels in couples who still wish to try to conceive naturally but are facing infertility. This gap in current literature means that there is an unmet need to understand the impact of infertility treatments on people experiencing this burden. The present study was conducted to assess whether the use of home ovulation calculators affects the levels of self-reported stress, psychological well-being, and quality of life in people experiencing infertility. By combining and revising existing questionnaires, we created a brief, reliable, self-administered measure of sexual dysfunction among couples experiencing infertility.

## Materials and methods

Participants were recruited from the Desai Sethi Urology Institute and the Department of Obstetrics and Gynecology at the Jackson Health System at the University of Miami. The study aimed to include a total of 50 couples seeking infertility treatment for either male or female factors. Couples were invited to participate in the study and were provided with anonymous, self-reported questionnaires pertaining to ovulation tracking and sexual habits. Both partners were required to complete the questionnaires.

The survey questions used in this study were adapted from established scales including the IIEF, FSFI, and FPI. These questionnaires were chosen for their validated measures of sexual function, fertility-related stress, and fertility-related distress, respectively.

To be eligible for the study, couples had to be actively consulting for infertility and willing to complete the survey. Couples were excluded from the study if they had already completed fertility treatments due to positive pregnancy outcomes or if they had ceased seeking fertility treatment. Additionally, couples who were unable to provide informed consent were also excluded.

Completed surveys were collected and subjected to descriptive analysis. The data collected included demographic information such as age, the number of years the couple had been trying to conceive, and responses to questions related to stress levels and the use of ovulation calculators. The researchers compared the stress levels between couples who reported using ovulation calculators and those who did not in order to determine the impact of ovulation calculators on stress levels in couples seeking fertility.

Statistical analysis was conducted to examine the differences between the two groups. Independent sample t-tests or non-parametric tests, such as Mann-Whitney U tests, were used as appropriate to compare the stress levels of couples using ovulation calculators and those who did not. The significance level was set at P < 0.05 to determine statistical significance.

The final analysis included responses from 50 couples who met the inclusion criteria. The collected data, including demographic information and survey responses, were carefully analyzed to evaluate any significant differences between the two groups and draw conclusions regarding the impact of ovulation calculators on stress levels in couples seeking fertility.

## Results

A total of 50 couples met the inclusion criteria of this study. Of these couples, the primary participant tended to be male (n = 38). The median age range for both male and female participants was between 35 and 39 years old. Thirty-seven couples reported tracking their ovulation status, with 25 of them using an ovulation calculator, six using predictor kits, and the rest using other methods (Table 1).

**Table 1:** Patient Demographics

Patient demographics		Total couples (N = 50)	Percentages
Gender (male)		38	76.9%
Male age	>50	1	3.8%
	40-49	9	23.1%
	35-39	16	38.5%
	30-34	11	30.8%
	25-29	1	3.8%
Female age	>50	1	3.8%
	40-49	10	26.9%
	35-39	9	23.1%
	30-34	13	34.6%
	25-29	1	3.8%
	>25	3	7.7%
Time spent trying to conceive	Less than a year	19	38.5%
	More than one year but less than three years	19	38.5%
	More than three years	3	7.7%
	More than five years	7	15.5%
Tracking ovulation using any method		7	15.5%
Using an ovulation calculator		36	73.1%
Using an ovulation predictor kit		25	50%

A sizable portion of couples (38.5%) had been trying to conceive for less than a year; a similar percentage (38.5%) had been trying for an average of more than one year but less than three years. Only 8% of patients had been undergoing fertility evaluations and treatments for more than three years. Surprisingly, 15% of respondents had been trying to conceive for more than five years.

Whether or not they were using ovulation calculators, females scored similarly in the four variables of the FSFI, including arousal, orgasm, satisfaction, and lubrication. When evaluating IIEF scores for male erectile function, the average score of males tracking ovulation was 12.0 ± 4.8, compared to 11.5 ± 5.4 in male patients who were not (P = 0.81). We observed that female sexual function and male erectile function scores did not differ between couples who track ovulation using a calculator and those who do not (Table 2).

**Table 2:** Positive Sexual Function Scores by Ovulation Calculator Status

Couples not using ovulation calculator (N = 14)				Couples using ovulation calculator (N = 36)		P-value
Female sexual function	Arousal	4.0	± 1.2	4.5	± 0.5	0.14
	Orgasm	4.4	± 0.8	4.2	± 0.9	0.47
	Satisfaction	4.6	± 0.6	3.8	± 1.3	0.06
	Lubrication	3.2	± 2.0	2.5	± 2.1	0.35
Male erectile function		12.0	± 4.8	11.5	± 5.4	0.81

**Figure 1:**
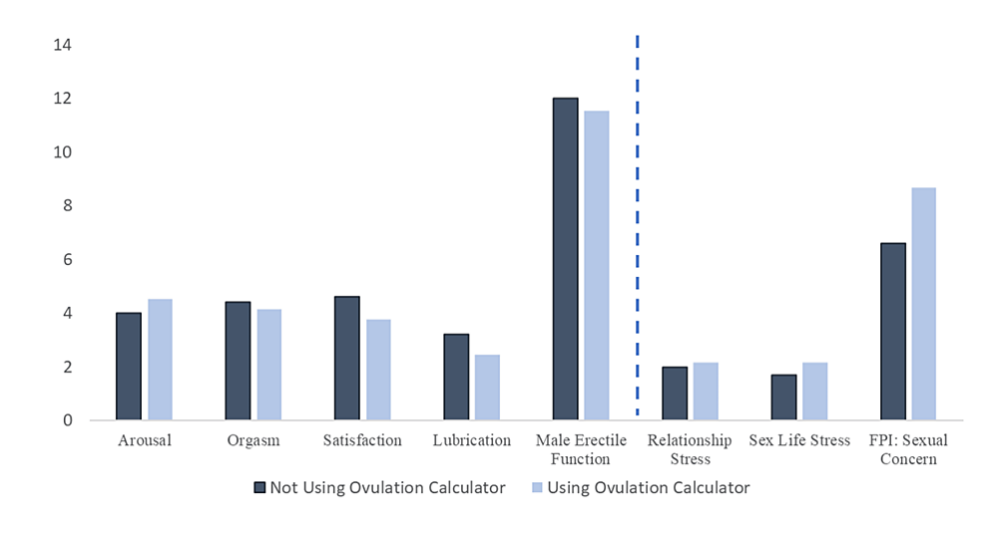
Representation of Positive and Negative Sexual Outcomes Questions measuring positive sexual outcomes: arousal, orgasm, satisfaction, and lubrication (maximum score: 5) Male erectile function score (maximum score: 15) Questions measuring negative sexual outcomes: relationship stress and sex life stress (maximum score: 5) FPI (sexual concern) (maximum score: 16) FPI: Fertility Problem Inventory

Similarly, both groups report similar levels of fertility-related stress impacting their relationship and sex life. However, couples who track ovulation had a higher FPI score in the domain of sexual concern (Table 3, Figure 1).

**Table 3:** Negative Outcome Scores by Ovulation Calculator Status

Couples not using ovulation calculator (N = 14)			Couples using ovulation calculator (N = 36)		P-value
Fertility Problem Inventory (sexual concern)	6.6	± 3.0	8.7	± 4.0	0.14
Negative impact of tracking ovulation on relationship	2.0	± 1.1	2.2	± 1.0	0.72
Negative impact of tracking ovulation on sex life	1.7	± 0.9	2.2	± 0.9	0.22

## Discussion

Natural pregnancy is most likely to occur during the fertile window, defined as three to five days before ovulation until the actual day of ovulation [11]. Nonetheless, there is a high degree of intra- and inter-individual variation in females' fertile window [12]. Timed intercourse (TI) during the fertile window increases the probability of conception, and ovulation calculators have been shown to be effective in synchronizing these two events [11,13]. This has led to a significant increase in the number of females' health and pregnancy applications, particularly for infertile couples, which now make up 7% of the applications market [14]. However, TI planned to coincide with ovulation has also been associated with stress and sexual dysfunction, which themselves can further impact fertility [15]. This study aimed to evaluate the association between the use of ovulation calculators for TI in couples seeking fertility and their impact on sexual function and fertility-related stress.

The unreliability and inaccuracies of ovulation calculators may contribute to the stress of their use. It is challenging to study the calculators' accuracy and impact on couples' fertility and induced stress as there are many heterogeneous products available [16]. Despite this, some studies have identified serious inaccuracies in most ovulation calculators as to the exact timing of the fertile window [16,17]. It has also been reported that inaccurate results in fertility awareness-based methods cause females to feel stressed, anxious, and worried, which can further impair fertility [16]. Nonetheless, two randomized controlled trials evaluating the stress levels, general health and well-being, and biochemical markers of stress among females seeking to conceive found that digital ovulation tests to schedule TI were not associated with any significant difference when compared to controls [3,18]. The discrepancy in results might be secondary to the type of ovulation calculator used, suggesting that future studies should compare different methods for tracking ovulation in relation to stress levels.

Stress is a psychological state accompanied by a physiological response, including a cortisol increase [19]. Cortisol is a well-known regulator of the hypothalamic-pituitary-gonadal axis and the sympathetic nervous system, and its elevation has been related to erectile and ejaculatory dysfunctions [19,20]. For instance, Bak et al. reported that as the number of TI increased, the number of males experiencing ED and extramarital sex also increased [20]. We found that couples using ovulation calculators for TI had higher scores for sexual dysfunction and a higher negative impact on their relationship and sex life; however, scores did not reach significance when compared to controls, probably due to the small sample size.

Infertility itself can impart significant stress in a couple's life. In females, it has been associated with poor marital adjustment and low quality of life, and in males, it has been related to less intercourse satisfaction [21]. Infertility can have a significant impact on male sexual dysfunction. The emotional and psychological stress associated with infertility can contribute to various sexual problems in males. The frustration and disappointment of being unable to conceive can lead to performance anxiety, reduced sexual desire, and erectile dysfunction. Males may experience feelings of inadequacy, guilt, and low self-esteem, which further exacerbate sexual difficulties [22]. Additionally, the pressure to perform sexually on specific fertile days can lead to increased stress and performance anxiety. The use of assisted reproductive technologies, such as in vitro fertilization, can also add to the strain on male sexual functioning. The constant focus on fertility treatments and the need to provide sperm samples can create feelings of pressure and negatively impact sexual function [22]. Communication issues between partners may arise, leading to decreased intimacy and strained relationships. It is essential for couples experiencing infertility-related sexual dysfunction to seek support, both individually and as a couple, through counseling, therapy, and medical interventions.

About 60% of the participants interviewed in our study had been trying to achieve a natural pregnancy for more than one year. The lack of differences observed between couples using and not using ovulation calculators could be explained by the fact that most of the participants had been seeking fertility for a long time and might have already been experiencing infertility-related sexual stress, resulting in a lack of increase or change from this baseline. Studies with larger cohorts are required to better understand and differentiate infertility-related stress from potential stress induced by ovulation calculators. The results of this study suggest that for couples undergoing fertility treatments, there may not be a significant difference in stress levels between those who use ovulation calculators and those who do not. However, it is important to note that these findings may have been influenced by the small sample size and limited statistical power, thus requiring further research with a larger sample size to provide a more robust conclusion.

Despite the limitations, this study contributes to the understanding of the impact of ovulation calculators on stress levels in couples seeking fertility and highlights the need for further investigation in this area. It also highlights the need for a better understanding of the sources of infertility-related stress and the development of effective interventions to support couples in this challenging experience. Further research is also necessary to fully understand the impact of infertility on couples and identify the most effective strategies for reducing stress and improving their overall well-being. Until then, it is important for healthcare providers to be aware of the potential for stress and provide support and resources to couples in their journey toward starting a family.

## Conclusions

The study's results revealed that the use of ovulation calculators did not lead to a statistically significant variance in stress levels between couples who employed them and those who did not. Consequently, it can be inferred that the integration of ovulation calculators into their fertility journey did not exert a significant impact on the stress levels encountered by these couples. However, the study highlights the significant impact of infertility-related stress on sexual function and mental health in both male and female partners. These results emphasize the importance of addressing the psychological aspects of infertility and providing comprehensive support to couples undergoing fertility treatments. These findings hold significance for both healthcare providers and couples as they contemplate the adoption of ovulation calculators as a means to facilitate conception. Further research is warranted to explore the complex interplay between ovulation calculator usage, infertility-related stress, sexual dysfunction, and mental health implications in couples seeking to conceive. Healthcare providers should consider incorporating mental health support into fertility treatment programs to optimize patient outcomes and overall well-being.
